# The Fundus Oculi

**Published:** 1919-11-08

**Authors:** 


### THE FUNDUS OCULI.

### A Review of Some Common Disorders.

Mr. W. H. McMullen, O.B.E., F.R.C.S., delivered a lecture on this subject on September 25 at the Royal Society of Medicine, in connection with the Fellowship of Medicine. The lecture took the form of a demonstration of a large series of coloured lantern slides. The following is a brief synopsis:

Before one can study disorders of the fundus oculi, it is necessary to become familiar with the very variable appearances presented by fundi which are normal.

In examining the fundus begin with the disc, for there many of the most important changes are found. Note first the colour. This is usually a pinkish hue, lighter than the surrounding fundus, but the depth of colour varies greatly in different individuals, and one should beware of diagnosing hyperæmia of the disc in the absence of other signs. In many discs, near the centre or towards the nasal side, there is a depression known as the physiological cup; this is produced by the arrangement of the nerve fibres coming through the lamina cribrosa. The floor of the cup is of lighter hue than the rest of the disc, and often presents a stippled appearance, caused by the meshwork of the lamina cribrosa. Note next the appearance of the margin of the disc. The normal margin is clearly defined, but it is not the same in all. Sometimes there is pigmented ring round it, often incomplete, the choroidal ring. In other cases the opening in the choroid is larger than that in the sclera and a light ring is visible, the sclerotic or connective tissue ring.

A slight striation of the disc and surrounding retina, and slight blurring of the disc margin, especially above and below, is fairly common, especially in hypermetropic eyes, and may lead to an erroneous diagnosis of optic neuritis.

Next note the vessels, starting from the disc and radiating outwards. Arteries may be distinguished from veins by their colour and by their size. The arteries are bright red, slightly smaller than the corresponding veins, and show a marked white central streak. This central streak is much more noticeable when the arterial walls are thickened by arterio-sclerosis. The veins are darker and more purple in colour, and the central white streak is less marked than in the arteries.

The general colour of the fundus shows a wide range of variation in different people, owing to the varying amounts of pigment in the pigmented retinal epitheleum and in the choroid. The development of pigment in the eye is usually proportional to that in the skin. In albinos pigment is wanting in the eye, the fundus is very pale, and the choroidal vessels are distinctly visible as red lines on a very light background. In many fair-complexioned people the appearance of the fundus approximates that seen in albinos, especially in the periphery. In some eyes an opposite appearance is produced by deep pigmentation of the stroma of the choroid, rendering the choroidal vessels visible as light-red lines on a darker background (choroid tigrée). This condition is frequently mistaken by beginners for choroiditis.

The appearances presented by the macula are likewise very variable. Sometimes it is difficult to make out the macula as distinct from the rest of the fundus. In an eye having a fair quantity of pigment the region of the macula is usually distinctly darker than the rest of the fundus. Sometimes one sees a faint white line or zone of slight striation surrounding the macula.

A fairly common physiological abnormality is that in which there is a very wide cup on one side of the disc, the nerve fibres being crowded into a crescentic area on the opposite side of the disc. Sometimes there is a white crescent (congenital crescent or Fuchs' coloboma) at the lower or lower and inner margin of the disc. These appearances probably result from some delay in the closure of the focal cleft.

In optic neuritis of moderate degree the margin of the disc is blurred, and there is some swelling, which is shown by the difference in focus of the disc and the neighbouring retina. In more severe degrees swelling and blurring become greater; the vessels, especially the veins, become distended and tortuous, and the tortuosity may be not only in the plane of the retina but also in an antero-posterior direction; the surface of the disc may present a markedly striated appearance; and there are often fine, radiating, flame-shaped hæmorrhages on the disc and in the surrounding retina.

Increased intracranial pressure may cause intense optic neuritis without any serious visual defect. In such cases, therefore, mere sight-testing is useless. There must be a careful ophthalmoscopic examination.

In neuro-retinitis, such as may occur in cases of nephritis or diabetes, there are hæmorrhages and exudates in the retina, in addition to the changes in the disc. A very characteristic appearance in albuminuric retinitis is the presence of small white spots arranged in radiating lines about the macula.

Extreme swelling and blurring of the disc are seen in cases of thrombosis of the central vein of the retina. The veins are much distended and tortuous, and very numerous hæmorrhages are scattered over the fundus. Thrombosis may occur only in a branch of the central vein. The changes then appear only in the corresponding part of the fundus.

Glaucoma is a condition which it is most important to recognise. In chronic glaucoma the disc is pale and cupped. The cupping can be recognised by the difference in form of the vessels at the bottom of the cup and at its edge, and from the way in which the vessels bend over sharply at the margin of the disc.

